# Pediatric scaphoid fractures: predictors of surgery and fracture complications

**DOI:** 10.1007/s00256-025-05049-3

**Published:** 2025-10-22

**Authors:** Vandan S. Patel, Lewis Fanney, Carlos Yaya-Quezada, Shahwar M. Tariq, Robert D. Boutin, Apurva S. Shah, Jie C. Nguyen

**Affiliations:** 1 Department of Radiology, Section of Musculoskeletal Imaging, Children’s Hospital of Philadelphia, 3401 Civic Center Blvd, Philadelphia, PA 19104 USA; 2 Division of Orthopedic Surgery, Children’s Hospital of Philadelphia, Philadelphia, PA USA; 3 Eastern Virginia Medical School, Norfolk, VA USA; 4 Department of Radiology, Stanford University School of Medicine, Stanford, CA USA; 5 Perelman School of Medicine, University of Pennsylvania, Philadelphia, PA USA

**Keywords:** Avascular necrosis, Complications, Imaging, Fracture, Osteonecrosis, Pediatric, Radiographs, Scaphoid, Skeletal maturity, Treatment

## Abstract

**Objectives:**

To identify clinical and radiographic findings of pediatric scaphoid fractures that predict the need for surgical treatment.

**Methods:**

A retrospective review of pediatric patients (≤ 18 years) with scaphoid fractures, who underwent radiographic examination and treatment at our tertiary care pediatric hospital, between 2018 and 2024, identified all surgically treated patients. From the remaining conservatively treated patients, age matched comparisons were randomly selected. After randomization and blinded to outcome, skeletal age, fracture characteristics (location, displacement, comminution, articular involvement, perifracture radiodensity, lobulated perifracture resorption, fracture gap), and presence or absence of osteonecrosis were recorded. Findings were compared between surgically treated and conservatively treatd groups to identify predictors of surgery.

**Results:**

Ninety-six children (81 males, 15 females, mean age: 15.0 ± 1.8 years, range: 11.0–17.8) included 48 in the surgery and 48 in the non-surgery groups. Proximal pole fractures (8.3%, 8/96), perifracture radiodensity (26.0%, 25/96), and presence of osteonecrosis (12.5%, 12/96) were uncommon, but were only found among patients in the surgery group. Presence of displacement (81.3% vs. 12.2%, *p* < 0.01) and more severe displacement (2.2 vs. 0.7 mm, *p* < 0.01), articular involvement (37.5% vs. 8.3%, *p* < 0.01), and lobulated perifracture resorption (64.6% vs. 10.4%, *p* < 0.01) were more common among patients in the surgery than patients in the non-surgery groups. Logistic regression analyses found proximal pole fractures (OR = 6.67, 95% CI: 1.48–16.78, *p* = 0.04), fracture displacement (OR = 6.30, 95% CI: 1.32–33.87, *p* < 0.01), and longer delay to initial radiographs (OR = 1.08, 95% CI: 1.04–1.18, *p* = 0.01) were independent predictors of surgery.

**Conclusion:**

Children with scaphoid fractures are more likely to require surgery if radiographic evaluation is delayed, fracture is displaced, or involves the proximal pole.

## Introduction

Scaphoid fractures account for over half of all carpal fractures [1–3]. Among adults, delayed diagnoses or suboptimally immobilized scaphoid fractures increase the risk for fracture nonunion, osteonecrosis, and development of carpal instability that can lead to premature osteoarthritis and long-term morbidity [4, 5]. Among children, most scaphoid fractures achieve osseous union following a trial of cast immobilization and only a minority require surgical intervention [6, 7]. This observation could be attributed to the presence of a vascularized chondroepiphysis of the skeletally immature scaphoid that facilitates spontaneous healing and remodeling [8]. Moreover, no prior study has investigated the association between surgery and fracture location, which can be a helpful radiographic finding that can guide clinical decision-making.

Although computed tomography (CT) and/or magnetic resonance imaging (MRI) are often utilized for surgical planning, radiographs remain the most frequently obtained imaging modality for the initial identification and serial monitoring of scaphoid fractures to directly influence clinical decision-making. To date, there is a paucity of published literature that exclusively examines factors that predict surgical management among children with scaphoid fractures. Thus, the purpose of this study was to examine clinical and radiographic features of pediatric scaphoid fractures that predict the need for surgical treatment.

## Methods

### Study group

**Fig. 1:**
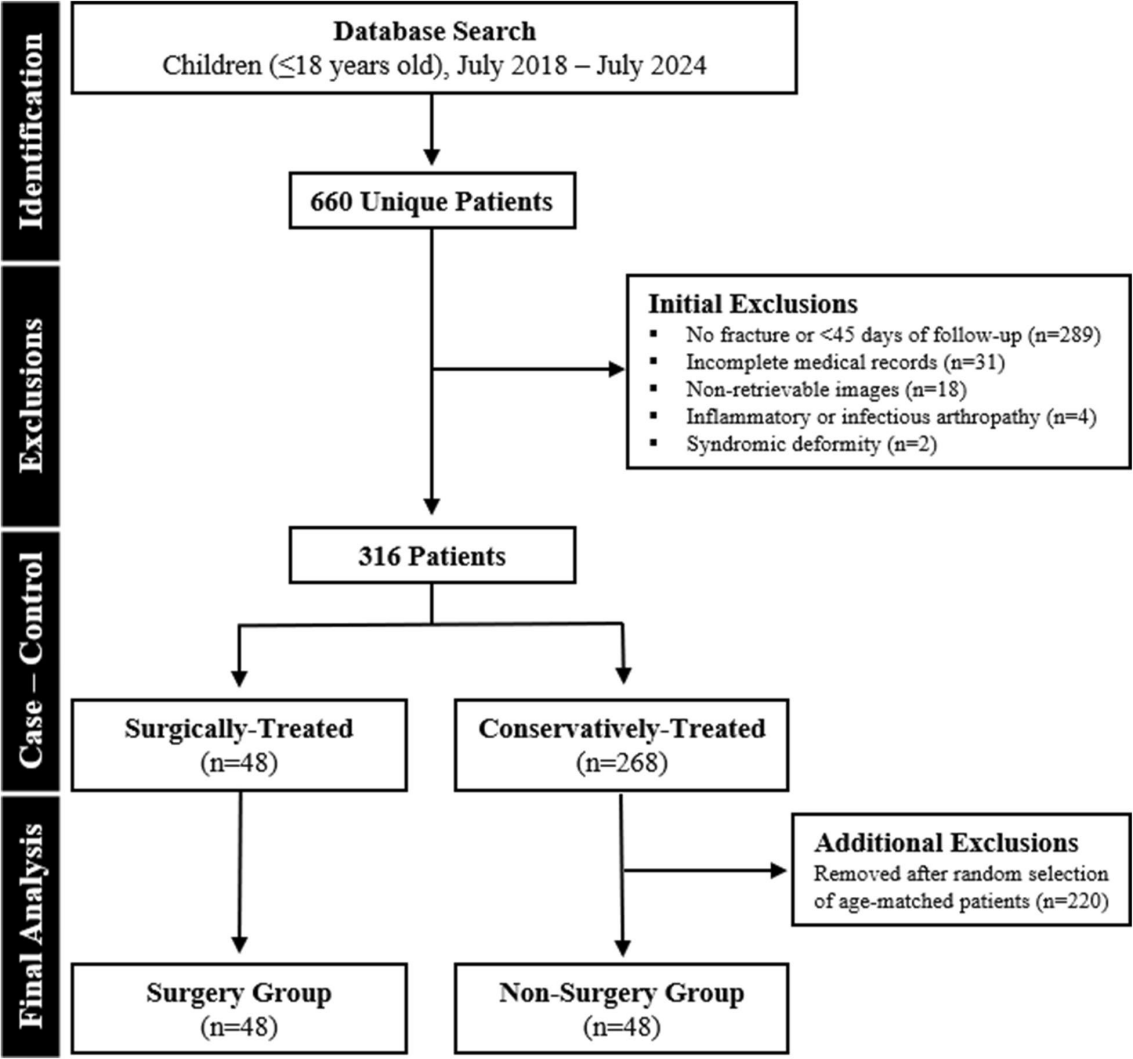
Flowchart outlines patient selection

Our study was approved by our Institutional Review Board (IRB) and performed in compliance with Health Insurance Portability and Accountability Act (HIPAA) regulations and with a waiver of written informed consent. A retrospective review of the electronic health record (Illuminate; Softek Illuminate, Overland Park, KS) was performed using the search term “scaphoid fracture” and setting the search to all radiographs of the hand or wrist, which identified 660 total pediatric patients (≤ 18 years) who underwent treatment at our tertiary care children’s hospital within the past 6 years (July 2018–June 2024). Patients were excluded if they met any of the following criteria: no scaphoid fracture or less than 45 days of clinical follow-up (*n* = 289), inadequate or incomplete medical records (*n* = 31), no retrievable initial radiographs within our institutional PACS system (Visage Imaging, Inc.; version 7.1.18; San Diego, CA) (*n* = 18), inflammatory or infectious arthropathy (*n* = 4), or syndromic deformity (*n* = 2). Among the remaining patients, 48 underwent surgical treatment, which provided > 90% power for detection of statistical significance (*p* < 0.05) [9]. From the remaining patients who underwent successful conservative treatment (defined by resolved pain, full range of motion, and osseous bridging with resolving or resolved fracture line), age-matched patients were randomly selected, comprising the non-surgery group (Fig. 1).

### Radiographic examinations

Hand and wrist radiographs were performed using direct digital radiography (Luminos Agile; Siemens, Erlangen, Germany) according to our institutional preset imaging parameters in order to generate the best tissue contrast using the lowest possible radiation dose. Standard patient positioning was used, and up to four views were obtained, which included posteroanterior (PA), oblique, lateral, and PA scaphoid views [10].

### Image review

After randomization and blind to outcome, a board-certified radiologist with fellowship training in pediatric and musculoskeletal radiology and 11 years of post-training clinical experience characterized all PA radiographs to determine the skeletal age, using the Greulich and Pyle classification [11]. Two readers, a fourth-year medical student with over 3 years and a third-year medical student with over 2 years of musculoskeletal research experience, underwent training with the pediatric musculoskeletal radiologist on the identification of fracture location and quantification of fracture displacement. Interrater reliability was performed utilizing 20% of the total cases.

**Fig. 2:**
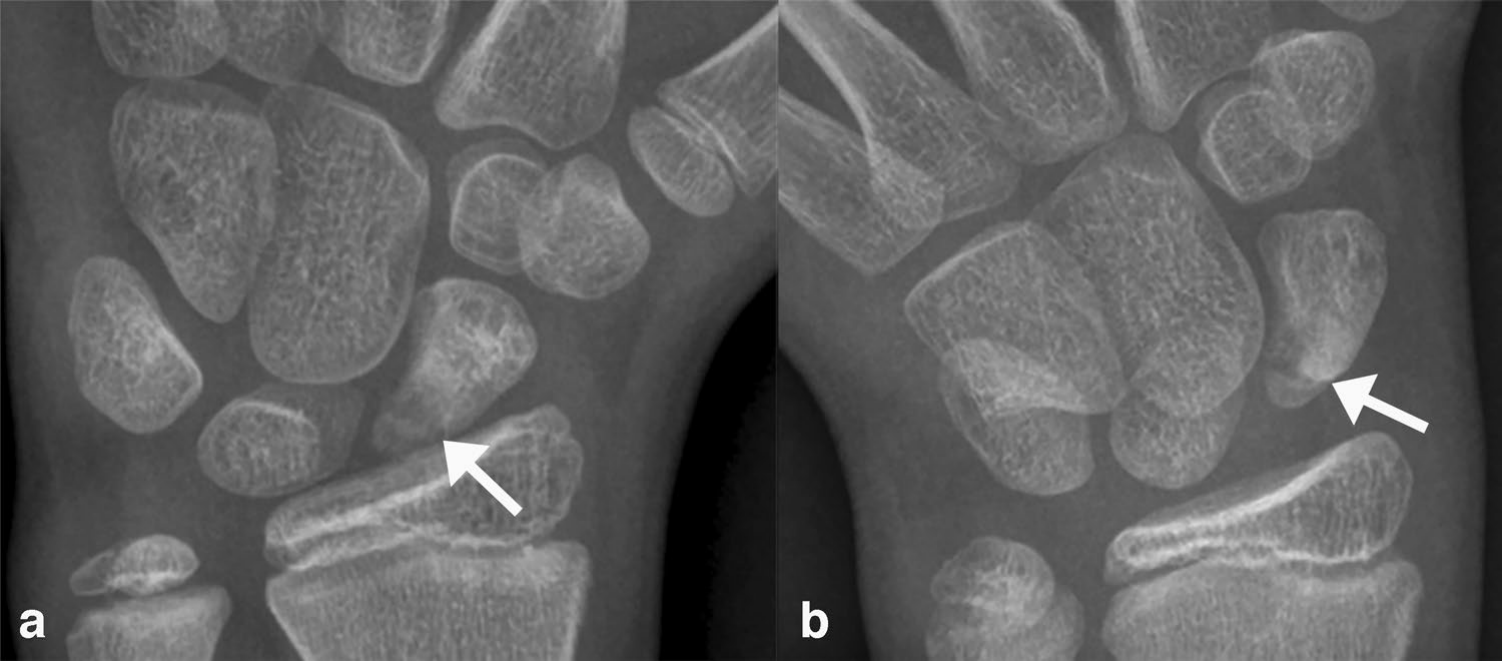
Intraarticular scaphoid fracture in a skeletally immature 11-year-old boy. Posteroanterior (PA, **a**) and PA scaphoid (**b**) radiographic views show a fracture (arrows) of the proximal pole that extends into the radioscaphoid joint. The patient underwent open reduction and internal fixation (ORIF) with a distal radius autograft

**Fig. 3:**
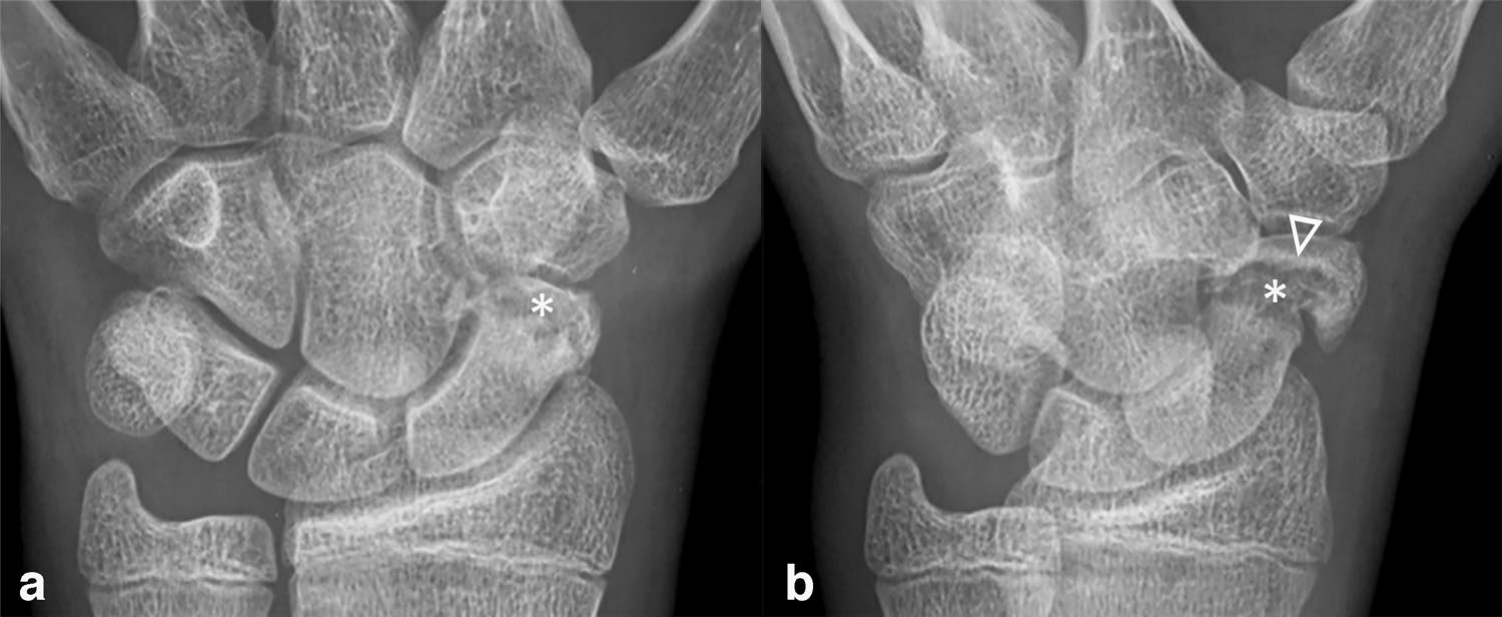
Perifracture findings in a skeletally immature 15-year-old boy. PA (**a**) and oblique (**b**) radiographic views show a fracture of the distal pole with lobulated resorption (asterisks) and perifracture radiodensity (triangle). The patient underwent ORIF with an iliac crest autograft

**Fig. 4:**
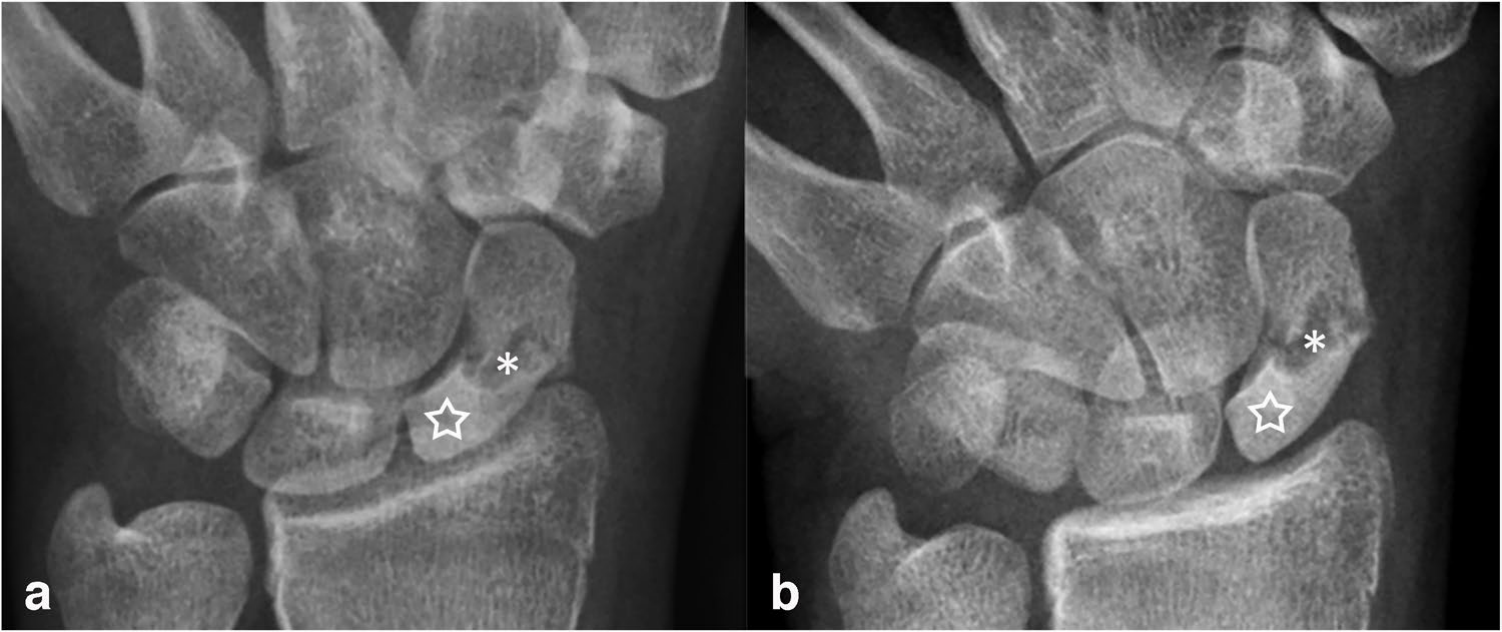
Osteonecrosis of the proximal scaphoid in a skeletally mature 15-year-old girl. PA (**a**) and PA scaphoid (**b**) radiographic views show a waist fracture with lobulated resorption (asterisks) and increased radiodensity of the proximal scaphoid (stars). The patient underwent ORIF with an iliac crest autograft

The pediatric musculoskeletal radiologist independently and retrospectively reviewed all studies to characterize fracture comminution, articular extension, perifracture radiodensity, lobulated perifracture resorption, and the presence or absence of osteonecrosis. Because the immature scaphoid ossification changes with progressive maturation, recognized landmarks in skeletally mature scaphoid may not be radiographically visible on all immature scaphoids. Thus, in keeping with the published literature, the location of the fracture was simply divided into those that involved the distal, middle, and proximal thirds of the scaphoid. Displacement was defined as present when cortical malalignment measured > 1 mm on any radiographic view [12], and using digital calipers, the largest displacement in millimeters was recorded. Fracture was comminuted if it contained > 2 fragments. Articular involvement was defined as present if the fracture cleavage plane extended into the radioscaphoid joint proximally or into the scaphoid-trapezium joint distally [13] (Fig. 2). Perifracture radiodensity and lobulated resorption were defined as increased radiodensity and concavities, respectively, along the fracture margin [14] **(**Fig. 3**)**. Finally, osteonecrosis was defined as diffuse increased radiodensity of the proximal fractured fragment(s) compared to the uninjured capitate [15] (Fig. 4).

### Clinical findings and outcome

Demographic data (chronological age, sex, and body mass index [BMI] percentile), injury mechanism (laterality, sports-related mechanism of injury, fall onto outstretched hand, etc.), days from injury to radiographic examination and to orthopedic referral, and outcomes were collected from the electronic medical records (EMR). Available weight and height (recorded within 6 months of clinical presentation) were used to calculate BMI percentile compared to children of the same age and sex using the Centers for Disease Control and Prevention 2000 growth chart [16]. A BMI of below the 5th percentile was considered underweight; between the 5th and 84th percentiles, normal or healthy weight; between the 85th and 94th percentiles, overweight; and above the 95th percentile, obese [17]. Delayed and non-union were defined clinically by the lack of fracture healing within and after 6 months, respectively [18]. Information pertaining to the surgical approach, the use of autograft, and harvest site were collected from the operative reports.

### Statistical analysis

Statistical analysis was performed with R and R Studio (version 1.1.423, R Foundation for Statistical Computing, Vienna, Austria), and a *p* value of < 0.05 considered statistically significant. The Shapiro-Wilk test was used to determine the normality of the distribution. Continuous variables, if normally distributed, were presented as means and standard deviations (SD), and, if not, were presented as medians and interquartile range (IQR). Categorical variables were presented as counts and percentages. Interrater agreement was assessed using the Cohen kappa coefficient for categorical variables, categorized as slight (≤ 0.20), fair (0.21–0.40), moderate (0.41–0.60), substantial (0.61–0.80), near perfect (0.81–0.99), or perfect (1.00), or using the intraclass correlation for continuous variables, categorized as poor (< 0.5), moderate (0.5 to < 0.75), good (0.75 to < 0.9), and excellent (0.9–1.0) [19, 20]. A Student’s *t*-test and Mann-Whitney *U* tests were used to compare continuous demographic and radiographic parametric and non-parametric variables, respectively. Chi-square tests were performed to compare categorical demographic data and radiographic parameters. Univariable and multivariable logistic regression analyses were performed to determine the utility of clinical and radiographic findings for predicting subsequent surgical intervention. Each test was chosen according to the type and distribution of these variables, findings, and number of groups.

## Results

Among 316 scaphoid fractures that met inclusion and exclusion criteria, 48 (15.2%) underwent surgical treatment. Thus, this study included a total of 96 children (mean age: 15.0 ± 1.8 years, range: 11.0–17.8), 48 (40 males, 8 females; mean chronological age: 15.1 ± 1.8 years, range: 11.0–17.7) in the surgery and 48 (41 males, 7 females; mean chronological age: 14.9 ± 1.8 years, range: 11.0–17.8) in the non-surgery groups. Fifty-four (56%) radiographic examinations included all 4 views and fourty-two (44%) included 3 views (PA, oblique, and lateral). All patients within the surgery group underwent open reduction and internal fixation (ORIF), 38 (79.1%) with and 10 (20.8%) without autograft (20 from iliac crest, 17 from distal radius, 1 from medial femoral condyle).

Table 1 summarizes the demographics and clinical data. Although chronological age was matched between the groups, patients within the surgery group had a more mature skeletal age compared to patients within the non-surgery group (mean: 16.0 vs. 15.0 years, *p* = 0.03). There were also longer delays among patients in the surgery than in the non-surgery groups in terms of days between injury and initial radiographs (median: 30.5 vs. 1.0 days, *p* < 0.01), between injury and orthopedic referral (median: 45.5 vs. 4.5 days, *p* < 0.01), and follow-up duration (125.0 vs. 56.0 days, *p* < 0.01). Patients in the surgery group also had higher rates of delayed healing and non-union when compared to the non-surgery group (91.7% vs. 4.2%, *p* = 0.01). No significant differences were found between the groups in terms of sex (*p* = 1), laterality (*p* = 0.54), mechanism of injury (*p* = 0.39), and BMI percentile (*p* = 0.79).

**Table 1:** Demographic and clinical data on pediatric patients with scaphoid fractures between surgery and non-surgery groups

Characteristics	Surgery group (*n* = 48)	Non-surgery group (*n* = 48)	*p*
Chronological age: mean ± SD (range) in years	15.1 ± 1.8 (11.0–17.7)	14.9 ± 1.8 (11.0–17.8)	0.42
Skeletal age: mean ± SD (range) in years	16.0 ± 2.1 (9.0–18.0)	15.0 ± 2.0 (10.0–18.0)	**0.03**
Sex: *n* (%)			1
Male	40 (83.3)	41 (85.4)	
Female	8 (16.7)	7 (15.6)	
Laterality: *n* (%)			0.54
Left	21 (43.8)	25 (52.1)	
Right	27 (56.2)	23 (47.9)	
Mechanism of injury: *n* (%)			0.39
Sports-related FOOSH^a^	34 (70.8)	28 (58.3)	
Non-specified FOOSH	13 (27.1)	18 (37.5)	
Unknown	1 (2.1)	2 (4.2)	
BMI percentile: *n* (%)			0.79
Underweight	0 (-)	1 (2.1)	
Normal	38 (79.2)	39 (81.2)	
Overweight or obese	10 (20.8)	8 (16.7)	
Days between: median (IQR)			
Injury and initial radiograph	30.5 (1.5–119.3)	1.0 (0.0–2.8)	**< 0.01**
Injury and orthopedic presentation	45.5 (13.8–133.8)	4.5 (2.0–10.5)	**< 0.01**
Presentation and latest follow-up	125.0 (91.0–220.8)	56.0 (45.0–74.3)	**< 0.01**
Delayed healing or non-union	44 (91.7)	2 (4.2)	**0.01**

*p*-values in bold are significant (< 0.05)

*BMI* body mass index, *FOOSH* fall on outstretched hand, *IQR* interquartile range, *SD* standard deviation

^a^Breakdown of sports (number in surgery group, number in non-surgery group): basketball (3, 11), football (10, 1), boxing or wresting (8, 2), soccer (5, 4), snowboarding (3, 1), skateboarding (1, 2), baseball (0, 2), motorized biking (0, 2), gymnastics (1, 1), lacrosse (1, 1), hockey (1, 0), skiing (1, 0), tennis (0, 1), and track (1, 0)

**Fig. 5:**
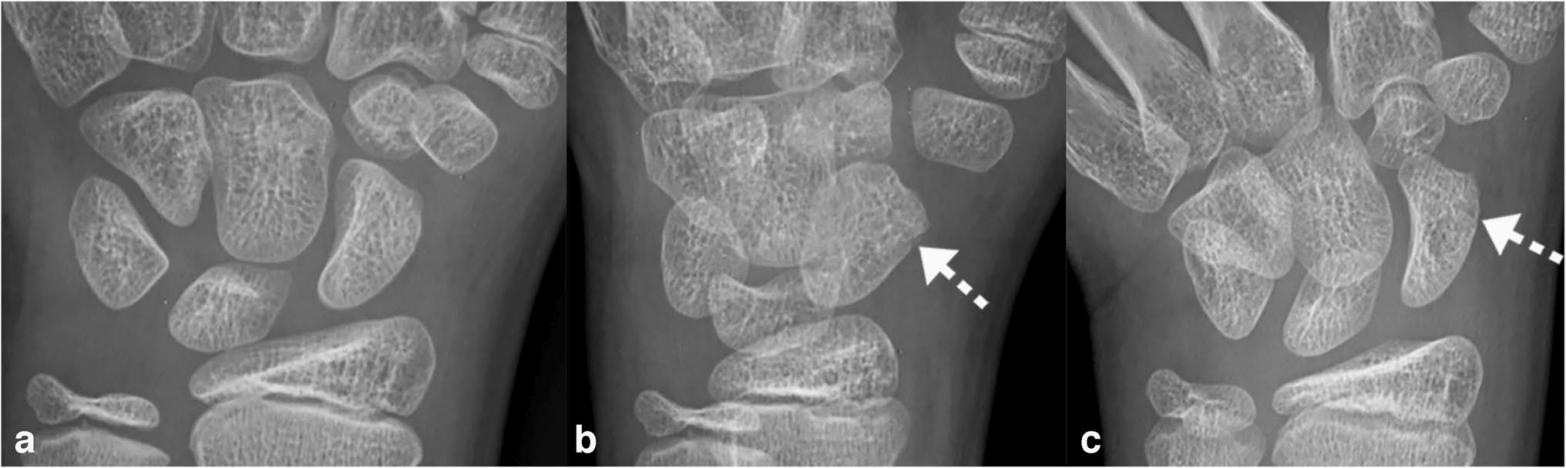
Distal pole fracture in a skeletally immature 11-year-old boy. PA (**a**), oblique (**b**), and PA scaphoid (**c**) radiographic views show a non-displaced fracture (dashed arrows) without articular involvement or perifracture findings. The patient was successfully treated with conservative treatment

Table 2 summarizes the various clinical and radiographic findings and interrater reliability. Interrater reliability was near perfect for fracture location (*κ* = 0.84), fracture displacement (*κ* = 0.89), and good for measured displacement (ICC = 0.83). Proximal pole fractures (8.3%, 8/96), perifracture radiodensity (26.0%, 25/96), and osteonecrosis (12.5%, 12/96) were uncommon, but only occurred among patients in the surgery group. Patients in the surgery group were also more likely to present with fracture displacement (81.3% vs. 12.2%, *p* < 0.01), have greater displacement (mean: 2.2 mm vs. 0.7 mm, *p* < 0.01), articular involvement (37.5% vs. 8.3%, *p* < 0.01), and lobulated perifracture resorption (64.6% vs. 10.4%, *p* < 0.01) compared to patients in the non-surgery group (Fig. 5).

**Table 2:** Radiographic findings among pediatric patients with scaphoid fractures

Characteristics	Surgery group (*n* = 48)	Non-surgery group (*n* = 48)	*p*	*κ/*ICC
Location of fracture: *n* (%)			** < 0.01**	0.84
Proximal pole	8 (16.7)	0 (-)		
Waist	36 (75.0)	37 (77.1)		
Distal pole	4 (8.3)	11 (22.9)		
Displacement: *n* (%)	39 (81.3)	6 (12.2)	** < 0.01**	0.89
Displacement: mean ± SD (range)	2.2 ± 1.1 (0.7–4.9)	0.7 ± 0.4 (0.1–1.8)	** < 0.01**	0.83
Comminuted fracture: *n* (%)	6 (12.5)	3 (6.3)	0.47	N/A^a^
Articular involvement: *n* (%)	18 (37.5)	4 (8.3)	** < 0.01**	N/A^a^
Perifracture radiodensity: *n* (%)	25 (52.1)	0 (-)	** < 0.01**	N/A^a^
Lobulated perifracture resorption: *n* (%)	31 (64.6)	5 (10.4)	** < 0.01**	N/A^a^
Widest fracture gap on any view: mean ± SD (range) in mm	4.3 ± 2.3 (1.2–9.6)	3.0 ± 1.9 (0.0–5.6)	0.09	N/A^a^
Osteonecrosis: *n* (%)	12 (25.0)	0 (-)	** < 0.01**	N/A^a^

*p*-values in bold are significant (< 0.05)

*SD* standard deviation

^a^Lacks independent assessments

Table 3 summarizes the logistic regression models for predictors of surgery. While delayed time to initial radiographs, proximal pole fractures, fracture displacement, articular involvement, perifracture radiodensity, lobulated resorption, and osteonecrosis reached statistical significance in the univariable analyses, in the multivariable analyses, only proximal pole fractures (OR = 6.67, 95% CI: 1.48–16.78, *p* = 0.04), fracture displacement (OR = 6.30, 95% CI: 1.32–33.87, *p* < 0.01), and delayed time to initial radiographs (OR = 1.08, 95% CI: 1.04–1.18, *p* = 0.01) remained statistically significant as independent predictors of surgery.

**Table 3:** Univariable and multivariable logistic regression models for predictors of surgery

Variables	Levels	Univariable model		Multivariable model	
		OR (95% CI)	*p*	Adjusted OR (95% CI)	*p*
Chronological age	Per-1 year increase	1.08 (0.86–1.36)	0.50	-	-
Sex	Female	Ref	Ref	-	-
	Male	1.22 (0.38–4.09)	0.74	-	-
Laterality	Left	Ref	Ref	-	-
	Right	1.45 (0.65–3.25)	0.36	-	-
BMI percentile	Underweight or normal	Ref	Ref	-	-
	Overweight or obese	1.28 (0.46–3.68)	0.64	-	-
Mechanism of injury	Non-sports related	Ref	Ref	-	-
	Sports related	1.79 (0.77–4.22)	0.18	-	-
Skeletal age	Per-1 year increase	1.18 (0.97–1.46)	0.10	-	-
Delay between injury and initial radiographs	Per day increase	1.11 (1.05–1.20)	** < 0.01**	1.08 (1.04–1.18)	**0.01**
Fracture location	Waist	Ref	Ref	-	**-**
	Proximal	8.00 (1.37–152.23)	**0.03**	6.67 (1.48–16.78)	**0.04**
	Distal	0.36 (0.09–1.17)	0.11	-	-
Fracture displacement	Absent	Ref	Ref	-	-
	Present	31.11 (10.92–104.13)	** < 0.01**	6.30 (1.32–33.87)	** < 0.01**
Comminuted fracture	Absent	Ref	Ref	-	**-**
	Present	2.08 (0.52–10.38)	0.32	-	**-**
Articular involvement	Absent	Ref	Ref	-	**-**
	Present	6.41 (21.12–23.76)	** < 0.01**	1.29 (0.08–39.04)	0.86
Perifracture radiodensity	Absent	Ref	Ref	-	**-**
	Present	49.02 (9.47–901.01)	** < 0.01**	16.19 (0.99–633.07)	0.06
Lobulated perifracture resorption	Absent	Ref	Ref	-	**-**
	Present	13.21 (4.92–40.39)	** < 0.01**	2.50 (0.38–15.66)	0.32
Osteonecrosis	Absent	Ref	Ref	-	**-**
	Present	25.01 (4.81–461.11)	** < 0.01**	0.38 (0.02–12.93)	0.54

*p*-values in bold are significant (< 0.05)

*BMI* body mass index, *CI* confidence interval, *OR* odds ratio

## Discussion

The most important finding of our study included more mature skeletal age among children in the surgically than non-surgically treated groups despite matched chronological age between the groups. Delay in initial radiographic assessment was also more common among children in the surgery than non-surgery groups. Radiographic findings of proximal pole fractures and fracture displacement were independent predictors of surgical intervention.

Our results highlight the spectrum of radiographic findings that can be associated with pediatric scaphoid fractures, adding to the current literature and helping to identify those findings that associate with delayed healing and the need for surgical intervention. Specifically, in adults, perifracture resorption, in the absence of bridging callus, results in a widened fracture gap and hinders healing [21], and increased radiodensity of the fractured fragment has been found to correlate with regional osseous necrosis on histopathology [15]. In our study, both perifracture resorption and perifracture radiodensity were significantly more common among children in the surgery than in the non-surgery group, but neither was an independent predictor of surgery. Rather, more diffuse radiodensity of the proximal scaphoid, as well as the presence of fracture displacement and delayed presentation to initial radiographs were independent predictors of surgery. These findings have not been previously investigated in children [22] and emphasize the critical role of radiographs in patient management and clinical decision-making.

Published literature on scaphoid fractures has predominantly focused on adults with a relative paucity of data on children. A recent systematic review that examined non-union of pediatric scaphoid fractures found a mean age at presentation between 12.2 and 15.3 years and no patient under 8 years [23]. This is concordant with the results of our study, with the youngest patient within the surgery group at 11 years. Moreover, the more advanced skeletal age of patients in the surgery than non-surgery groups gathers evidence on the critical role of skeletal maturity on regional healing and remodeling potential. Scaphoid ossification becomes radiographically visible around 6 years of age, and it enlarges over time through progressive replacement of the peripherally located vascularized immature cartilage. Skeletal maturity is reached when the immature cartilage is completely replaced by bone, which occurs around 13 years of age in females and 15 years of age in males [24, 25]. It is postulated that this immature cartilage not only protects the underlying ossification center from injury but also facilitates spontaneous healing, which is diminished near and after skeletal maturity. The latter likely accounts for the relatively higher rates of failed non-surgical management among adults [12].

Although fractures of the distal scaphoid are more common among younger children, the vast majority of patients within our surgery group had fractures that involve the scaphoid waist, a more common pattern observed in adults [26, 27]. This may be explained by the normal direction of physiologic maturation of the scaphoid, which progresses from distal to proximal direction [24, 28]. In adult patients, high rates of complications have been attributed to injury to the nutrient vessels, which courses from the distal to the proximal scaphoid, predisposing the proximal pole to post-traumatic osteonecrosis [29]. Osteonecrosis of the proximal scaphoid was only observed among patients in the surgery group.

Our study has several limitations. The retrospective design of our study prevented the gathering of additional clinical information and additional images. However, we only included children with confirmed scaphoid fractures with a minimum of 45 days of clinical follow-up. This study was purposely designed to mimic routine clinical practice where delayed presentation for scaphoid fractures is not uncommon. Second, only a minority of children with scaphoid fractures required and underwent surgical intervention, due to the robust spontaneous healing potential of the immature skeleton. But our study remains the largest case-control study on pediatric scaphoid fractures, adding to our existing understanding of the various radiographic findings that can be observed with pediatric patients. Finally, the use of the term osteonecrosis based on radiographic findings of increased radiodensity has intrinsic limitations, because new bone formation, impacted trabeculae, and relative osteopenia of adjacent bone from immobilization can also produce asymmetrically increased radiodensity [30].

In conclusion, patients in the surgery group had more advanced skeletal age compared to the non-surgery group despite matched chronological age. Fractures involving the proximal pole, presence of perifracture radiodensity, and osteonecrosis were exclusively found in those within the surgery group. More importantly, for clinical providers and reading radiologists, late presentation for the initial radiographs, proximal pole fractures, and greater fracture displacement are independent predictors of surgery and thus should be reported and provided prompt orthopedic referral.

## Data Availability

Data is available upon reasonable request.
